# The Ever-Growing Puzzle of Asynchronous Release

**DOI:** 10.3389/fncel.2019.00028

**Published:** 2019-02-12

**Authors:** Andrei Rozov, Alexey P. Bolshakov, Fliza Valiullina-Rakhmatullina

**Affiliations:** ^1^Laboratory of Neurobiology, Institute of Fundamental Medicine and Biology, Kazan Federal University, Kazan, Russia; ^2^Department of Physiology and Pathophysiology, University of Heidelberg, Heidelberg, Germany; ^3^Institute of Higher Nervous Activity and Neurophysiology, Russian Academy of Sciences (RAS), Moscow, Russia; ^4^Laboratory of Electrophysiology, Pirogov Russian National Research Medical University, Moscow, Russia

**Keywords:** presynaptic, calcium, synaptotagmins, calcium extrusion, synaptic release

## Abstract

Invasion of an action potential (AP) to presynaptic terminals triggers calcium dependent vesicle fusion in a relatively short time window, about a millisecond, after the onset of the AP. This allows fast and precise information transfer from neuron to neuron by means of synaptic transmission and phasic mediator release. However, at some synapses a single AP or a short burst of APs can generate delayed or asynchronous synaptic release lasting for tens or hundreds of milliseconds. Understanding the mechanisms underlying asynchronous release (AR) is important, since AR can better recruit extrasynaptic metabotropic receptors and maintain a high level of neurotransmitter in the extracellular space for a substantially longer period of time after presynaptic activity. Over the last decade substantial work has been done to identify the presynaptic calcium sensor that may be involved in AR. Several models have been suggested which may explain the long lasting presynaptic calcium elevation a prerequisite for prolonged delayed release. However, the presynaptic mechanisms underlying asynchronous vesicle release are still not well understood. In this review article, we provide an overview of the current state of knowledge on the molecular components involved in delayed vesicle fusion and in the maintenance of sufficient calcium concentration to trigger AR. In addition, we discuss possible alternative models that may explain intraterminal calcium dynamics underlying AR.

## Introduction

**Figure 1:**
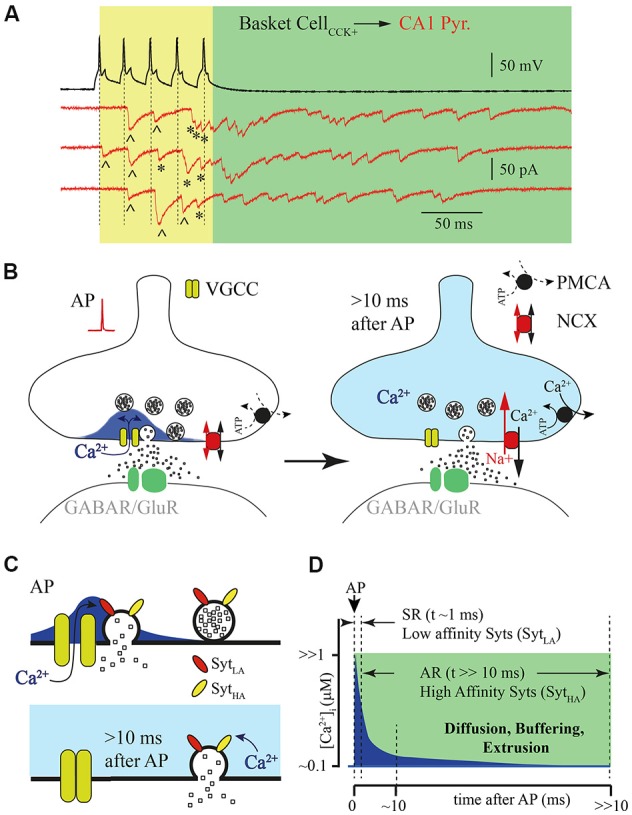
Asynchronous release (AR) is temporally separated from action potential (AP) generated calcium concentration microdomains. **(A)** Example traces of responses recorded from a pair of connected cells, a hippocampal presynaptic cholecystokinin (CCK)^+^ basket cell (black) and postsynaptic CA1 pyramidal neuron (red, three subsequently recorded traces). Five APs (50 Hz) trigger synchronized phasic IPSC [labeled with (^^^)] and delayed responses that can be observed both during the AP train [yellow window; labeled with (*)] and after termination of presynaptic stimulation (green window). **(B)** Schematic drawing of the presynaptic calcium concentration dynamics after a single AP. Opening of the voltage gated calcium channel (VGCC) causes formation of a calcium concentration microdomain—a short-lasting local elevation of [Ca^2+^]_i_ sufficient to trigger phasic release (left panel). After closure of the VGCC, [Ca^2+^]_i_ radially diffuses and equilibrates within the terminal and then further declines due to binding to endogenous buffers and extrusion (right panel). **(C)** Schematic drawing of vesicle fusion driven by an AP evoked Ca^2+^ micro/nano-domain (upper panel). Note that high synchrony arises from the low affinity of the Ca^2+^ sensor (Syt_LA_) and tight spatial coupling of the Ca^2+^ source and Ca^2+^ sensor. The lower panel shows delayed vesicle fusion mediated by residual [Ca^2+^]_i_ remaining in terminals several milliseconds after the last AP. In this case recruitment of high affinity synaptotagmins (Syt_HA_) is necessary, but vesicles can be spatially separated from VGCC, since release is triggered by bulk [Ca^2+^]_i_. **(D)** Schematic representation of the [Ca^2+^]_i_ time course at the release site (blue) after AP. Dotted lines show time windows for synchronous (SR) and AR components of release that are probably mediated by synaptotagmins with different Ca^2+^ affinities.

In most of the synapses in the central and peripheral nervous system, release of synaptic vesicles is tightly temporally coupled to presynaptic action potentials (APs). Usually synaptic delay, the time between the peak of the AP and the onset of the postsynaptic response, does not exceed a few milliseconds. This holds true even for synapses where APs can trigger multi vesicular release (Watanabe et al., 2005). Short synaptic delay also suggests a small range of synaptic jitter, which, in turn, provides the functional basis for the synchronization of postsynaptic responses (Burkitt and Clark, 1999). This feature of synaptic transmission allows rapid information transfer between connected cells and within neuronal networks. The level of synchronization between APs and synaptic vesicle fusion is mainly determined by the affinity of vesicular Ca^2+^ sensors and Ca^2+^ dynamics within presynaptic microdomains. In most cases, Ca^2+^ concentration collapses below the threshold level for triggering vesicle fusion within a few milliseconds of the AP reaching the presynaptic terminal. Generally, even high frequency bursts of APs result in highly synchronized postsynaptic activity. However, at some synapses high frequency stimulation can trigger not only synchronized phasic transmitter release but can also generate vesicle fusion that lasts for tens or hundreds of milliseconds after the end of the AP burst (Figure 1A; Hefft and Jonas, 2005; Daw et al., 2009; Ali and Todorova, 2010; Wen et al., 2010; Jappy et al., 2016; Chen et al., 2017; Li et al., 2017; Luo and Sudhof, 2017; Turecek and Regehr, 2018). This phenomenon is known as asynchronous release (AR). Most likely, this mode of release is not involved in rapid information transfer within CNS, but plays an important role in the generation of long-lasting forms of synaptic plasticity especially at those synapses where plasticity requires the involvement of extrasynaptic metabotropic receptors (Jappy et al., 2016). Also, taking into account that the output of synapses with AR lasts for tens of milliseconds after the end of the presynaptic AP burst, neurons possessing the ability to release neurotransmitter in this delayed asynchronous fashion may participate in the generation of low frequency oscillations. For instance, hippocampal cholecystokinin (CCK)-positive basket interneurons show prominent AR and play a key role in the maintenance of the hippocampal theta rhythm (Hefft and Jonas, 2005; Klausberger and Somogyi, 2008). Finally, similarly to phasic release, AR undergoes short-term plasticity dependent on presynaptic stimulation frequency and duration (Iremonger and Bains, 2007; Ali and Todorova, 2010). Thus, the understanding of mechanisms underlying AR will contribute to our knowledge on the generation of different forms of synaptic plasticity and network integration of distinct neuronal types.

Over the last couple of decades, the molecular components and mechanisms involved in delayed synaptic vesicle fusion have been extensively studied using different approaches. It has been proposed that the synchronous and asynchronous modes of release recruit different Ca^2+^ sensors and are differentially regulated by proteins involved in the vesicle cycle. Both release modes are Ca^2+^-dependent, however, the number of Ca^2+^ ions required to bind with Ca^2+^ sensors that is necessary to trigger these distinct types of release might be different. Another question that remains a subject of discussion is: what are the Ca^2+^ sources for triggering the two types of vesicle release? While there is a common agreement that synchronous release is mainly triggered by Ca^2+^ influx through presynaptic voltage-gated Ca^2+^ channels, the source of long-lasting Ca^2+^ entry required for AR triggering remains poorly identified and, probably varies depending on the identity of the presynaptic neuron. The main differences in mechanisms underlying phasic and asynchronous modes of release have been discussed in an excellent review by Kaeser and Regehr (2014). However, it is still unclear why the same stimulation protocol on two types of presynaptic inputs to the same postsynaptic cell triggers highly synchronized release from one type of terminal and strong long lasting AR at the other (Hefft and Jonas, 2005). In this review article, we discuss the molecular players involved in AR generation in inhibitory and excitatory central synapses and in neuromuscular junctions. In addition to that, we review the current opinion on the mechanisms that allow a sufficient level of presynaptic Ca^2+^ to trigger delayed fusion of synaptic vesicles. Finally, we suggest the possible involvements of calcium extrusion pumps in AR generation and maintenance.

## Presynaptic Calcium Domains and Asynchronous Release

According to the most widely accepted model, synaptic release is triggered in the active zones by Ca^2+^ entering through voltage gated calcium channels (VGCCs). When these channels open during AP, short lasting and spatially restricted elevation of intraterminal Ca^2+^ ([Ca^2+^]_i_) occurs in close vicinity to the channels or the cluster of the VGCCs known as nano- or microdomains (Chad and Eckert, 1984; Simon and Llinás, 1985; Neher, 1998; Eggermann et al., 2011). Although, direct measurements of [Ca^2+^]_i_ dynamics in the active zone are technically challenging at this time, all existing mathematical models predict that [Ca^2+^]_i_ elevation sufficient for triggering fast phasic release remains in the microdomain no longer than a few milliseconds after AP (Arai and Jonas, 2014). Then [Ca^2+^]_i_ equilibrates within the terminal because of radial diffusion, and further declines due to binding to endogenous buffers and calcium extrusion (Figures 1B,D). The rapid temporal dynamics of calcium concentration in microdomains ensures high fidelity AP-driven phasic synaptic transmission. However, it seems very unlikely that the same calcium sensor (synaptotagmins) and calcium source are involved in the generation of both the phasic and asynchronous components of evoked release. Taking into account the duration of AR, at some synapses hundreds of milliseconds, the [Ca^2+^]_i_ available at the release site should be substantially lower than that in the microdomain during phasic release. This assumption strongly suggests three possible scenarios for AR generation: (1) affinity of the calcium sensor mediating AR should be high enough so that fusion events may be triggered by the remaining bulk [Ca^2+^]_i_; (2) invasion of APs to the presynaptic terminals, besides opening VGCC, triggers additional long lasting Ca^2+^ entry; and (3) a combination of both high-affinity calcium sensors and an additional calcium source is necessary for AR generation.

## Calcium Sensors Underlying Asynchronous Release

One of the popular hypotheses to explain delayed vesicle fusion after high frequency presynaptic stimulation is that the synchronous and asynchronous modes of release are triggered by different types of Ca^2+^ sensors (Figure 1C). The selective suppression of AR by moderate concentrations of EGTA (Hefft and Jonas, 2005; Iremonger and Bains, 2007) speaks in favor of this notion, suggesting both spatial separation of the asynchronously released vesicles from Ca^2+^ microdomains and high affinity of the sensor mediating this mode of release. It is commonly accepted that the role of vesicular Ca^2+^ sensor is played by proteins belonging to the synaptotagmin (Syt) family consisting of 17 members. They differ in their expression pattern and Ca^2+^ binding properties (Bhalla et al., 2008; Gustavsson et al., 2008; Craxton, 2010; Moghadam and Jackson, 2013). The vital role in the generation of the phasic component of synaptic release is usually attributed to Syt1 and Syt2. Indeed, homozygous Syt1 knockout mice die within 48 h of birth. Analysis of synaptic transmission between cultured hippocampal pyramidal neurons has shown that deletion of Syt1 leads to the selective loss of fast evoked synaptic release while AR and spontaneous vesicle fusion remain unaffected (Geppert et al., 1994). Furthermore, point mutations in Syt1 that result in either reduction of Ca^2+^ affinity or a decrease in phospholipid binding also selectively suppress the phasic component of evoked release (Pang et al., 2006; Fleidervish et al., 2010). Similarly to Syt1 omission, knockout of Syt2 results in severe desynchronization of synaptic release from presynaptic APs (Sun et al., 2007). In calyx of Held synapses, synaptic delay in Syt2-knockout mice was approximately 3–4 times longer than synaptic delay in wild type animals. Moreover, in wild type calyxes, when [Ca^2+^]_i_ exceeded 1 μM most of the vesicles were released within first few milliseconds, resulting in fast rising excitatory postsynaptic currents (EPSCs); in knockout animals, using flash photolysis of caged Ca^2+^, release rate progressively increases reaching a peak about 100–200 ms after the flash that triggered Ca^2+^ uncaging (Sun et al., 2007). The authors concluded that the presence of Syt2 is essential for rapid synchronization of vesicle fusion at high [Ca^2+^]_i_. The major role of Syt1 and Syt2 as the vesicular calcium sensors at GABAergic and glutamatergic synapses was further proven in a number of studies (Xu et al., 2007; Südhof, 2013; Chen et al., 2017; Li et al., 2017). Of the synaptotagmins Syt1 and Syt2 have the lowest Ca^2+^ affinity (EC50 = 10–20 μM; Sugita et al., 2002). Therefore, although these Syts are suitable for triggering highly synchronized phasic release during the short-lived [Ca^2+^]_i_ elevation within the microdomain, it seems unlikely that they can maintain vesicle fusion even a few milliseconds after VGCC closure.

Another isoform of synaptotagmin, Syt7, that has been proposed to mediate AR, has tenfold higher Ca^2+^ affinity (EC50 = 1–2 μM; Sugita et al., 2002). At wild-type zebrafish neuromuscular junctions, high frequency stimulation leads to a high level of desynchronization of vesicle fusion that may be observed as a barrage of EPSCs between two subsequent APs. Knockdown of Syt7 almost completely abolished these events without having a major effect on the synchronous release occurring 1–3 ms after APs (Wen et al., 2010). Conversely, in a study conducted in T. Sudhof’s laboratory (Maximov et al., 2008) deletion of Syt7 did not have any effect on either synchronous or AR measured at inhibitory synapses in cortical neuronal cultures. However, in a subsequent study the same group used a knockdown approach to eliminate the contribution of Syt7 to AR and they found that indeed, similarly to the neuromuscular junction, this isoform of synaptotagmin plays a significant role in AR generation (Bacaj et al., 2013). The authors explained the apparent discrepancy between these two reports as possible developmental compensation in Syt7 knockout animals. Finally, involvement of the Syt7 isoform in delayed vesicle fusion during neuroendocrine exocytosis has been demonstrated in numerous studies (Sugita et al., 2001; Shin et al., 2002; Fukuda et al., 2004; Tsuboi and Fukuda, 2007; Gustavsson et al., 2008; Schonn et al., 2008; Gustavsson and Han, 2009; Li et al., 2009; Segovia et al., 2010). Recently, the crucial role of Syt7 in AR generation has been confirmed in inhibitory hippocampal synapses (Li et al., 2017), cerebellar GABAergic (Chen et al., 2017) and glutamatergic (Turecek and Regehr, 2018) synapses, and at excitatory calyx of Held synapse (Luo and Sudhof, 2017).

However, despite the growing data pool supporting the notion that Syt7 is the AR calcium sensor, there are several features of this isoform suggesting a more complex mechanism underlying AR. First, in contrast to Syt1 and Syt2, Syt7 was found on the presynaptic plasma membrane and other internal membranes, but not synaptic vesicles (Sugita et al., 2001; Virmani et al., 2003; Takamori et al., 2006; Südhof, 2013) implying that this isoform either does not participate in vesicle exocytosis or does it in a non-canonical fashion. An alternative function of Syt7 was proposed by Liu et al. (2014) and they demonstrated the involvement of Syt7 in synaptic vesicle replenishment in response to high frequency depleting stimulation. In support of this hypothesis it has been recently shown that robust high frequency stimulation (20 Hz for 5 s) promotes Syt7-dependent endocytosis and formation of Syt7-containing vesicles, which might be later released asynchronously (Liu et al., 2014). This mechanism may underlay the delayed release observed at the cerebellar GABAergic basket to Purkinje cell synapses, where AR may be triggered by several repetitions of 20 Hz 50 APs trains (Chen et al., 2017), but fails to explain how in the terminals of hippocampal CCK-positive basket interneurons a single burst of a few APs (3–5 APs at 50 Hz; Figure 1A) generates AR lasing over 100 ms (Hefft and Jonas, 2005; Daw et al., 2009; Ali and Todorova, 2010; Jappy et al., 2016).

Second, the main evidence that Syt7 is the AR calcium sensor comes from experiments conducted on knockout animals. Indeed, deletion of this isoform leads to a reduction of the delayed release component in synapses which have a moderate contribution of AR to synaptic response (Bacaj et al., 2013; Chen et al., 2017; Turecek and Regehr, 2018). Nevertheless, there is evidence that the “desynchronizing” action of Syt7 depends on the identity of the interaction partners in the SNARE complex. For instance, in hippocampal cultured neurons substitution SNAP-25 by SNAP-23 in the presence of endogenous Syt7 resulted in strong desynchronization of evoked release, but omission of Syt7 in SNAP-23 expressing cultures made release even more asynchronous (Weber et al., 2014). Moreover, Weber and co-authors showed that asynchronously released vesicles carried Syt1, but not Syt7. Finally, the single-cell expression profile of synaptotagmins clearly shows that Syt7 is expressed in most hippocampal and neocortical neurons (Zeisel et al., 2015) and the level of Syt7 mRNA in excitatory hippocampal cells, which show very moderate AR, is substantially higher than in CCK/CB1-positive interneurons, which demonstrate pronounced AR (Zeisel et al., 2018). Recent articles proposed that this isoform is responsible for paired pulse synaptic facilitation at a number of excitatory synapses (Jackman et al., 2016; Turecek and Regehr, 2018) assuming that recruitment of Syt7 in synaptic release occurs during the second AP. Nevertheless, most of these connections, three hippocampal and one corticothalamic, do not show AR at a level that might have physiological relevance. Thus, one can conclude that different isoforms of synaptotagmins can play different roles in determining the modality of vesicle fusion at different synapses. Most likely, low-affinity isoforms Syt1 and Syt2 are responsible for the high level of synchronization of fast release with presynaptic APs, while high-affinity Syt7 participates in generation of delayed release. However, the mechanism of recruitment of Syt7 in phasic and AR needs to be identified.

Doc2 proteins were proposed as another candidate for the Ca^2+^ sensor responsible for AR (Yao et al., 2011; Xue et al., 2015). However, the initial finding that knockout of cytosol soluble Doc2A reduces the asynchronous component of evoked release has not been confirmed by other groups (Groffen et al., 2010; Pang et al., 2011). More recently, it has been shown that proteins of the Doc2 family participate in spontaneous release rather than take part in AR generation (Ramirez et al., 2017). In particular, Doc2α is involved in spontaneous release at excitatory synapses while Doc2β knockout selectively affects spontaneous release from GABAergic terminals (Courtney et al., 2018). Finally, consideration of Doc2 proteins as specific Ca^2+^ sensors for AR is challenged by single-cell RNA-seq data, according to which expression of Doc2 proteins is substantially higher in excitatory hippocampal and cortical neurons with very weak AR than in those subpopulations of interneurons that have very pronounced AR (Zeisel et al., 2018).

## Calcium Sources for Triggering Asynchronous Release

Despite the fact that asynchronous and phasic releases can be triggered by distinct Ca^2+^ sensors, the main prerequisite for triggering prolonged delayed release is long-lasting presynaptic [Ca^2+^]_i_ elevation. Thus, the two main questions when studying AR are:

1. what is the source of presynaptic Ca^2+^ that triggers AR?
2. how does elevated [Ca^2+^]_i_ persist in presynaptic terminals for tens or hundreds of milliseconds?

**Figure 2:**
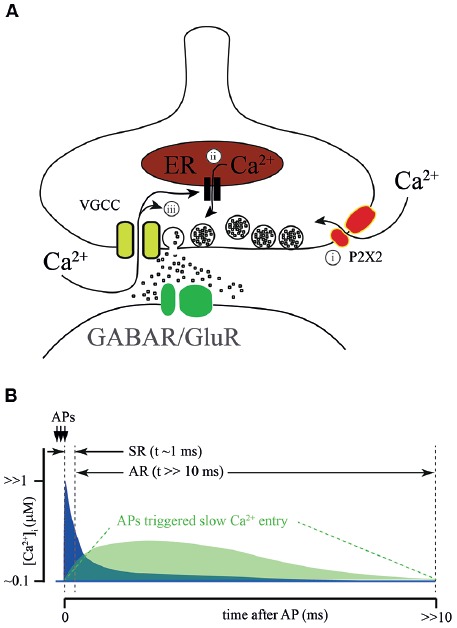
Sources of calcium for AR generation. **(A)** Proposed sources of calcium for AR generation: (i) ATP-gated ionotropic P2X2 receptors; calcium- dependent release from intracellular calcium stores; and (ii) calcium-dependent prolongation of calcium entry through VGCC. **(B)** Schematic representation of a hypothetical [Ca^2+^]_i_ time course at the release site during SR and AR. Conventional [Ca^2+^]_i_ elevation due to the flux through VGCC during AP (blue) combines with Ca^2+^ entry *via* an additional AP activated Ca^2+^ source (green). Dotted lines show time windows for SR and AR.

The suggested sources of Ca^2+^ for AR generation are summarized on Figure 2A. It has been proposed that P2X2 receptors mediate/modulate AR at glutamatergic synapses formed by Schaffer collaterals on CA1 stratum radiatum interneurons. Here a high frequency train of 3 or 9 stimuli triggered AR lasting for several seconds (Khakh, 2009). In half of the neurons tested, the frequency of the post-train asynchronous event could be reduced by application of a P2X2 antagonist, however, drug application did not have any effect on the remaining interneurons. This finding has potential interest for two major reasons. First, it gives a hint that the P2X2 receptor is expressed in the brain in adulthood which so far has not been shown by RNA-seq (Zeisel et al., 2015; Cembrowski et al., 2016). Second, it shows that activation of Ca^2+^-permeable purinergic receptors may play a modulatory role in transmitter release at glutamatergic synapses.

In frog neuromuscular junctions, long-lasting amplification of Ca^2+^ transients was suggested to be due to Ca^2+^-induced Ca^2+^ release from intracellular stores. In two articles published by Narita et al. in 1998 and 2000, the authors claimed that during high frequency stimulation [Ca^2+^]_i_ in motor neuron terminals reaches a sufficient level for the activation of ryanodine receptors located on presynaptic Ca^2+^ depots (Narita et al., 1998, 2000). Subsequently, this can trigger massive Ca^2+^ release from presynaptic intracellular stores. The major proof of this conclusion is that, in the presence of thapsigargin, the amplitude of the [Ca^2+^]_i_ transient evoked by high frequency afferent nerve stimulation was greatly reduced relative to control. The role of intracellular Ca^2+^ depots in shaping fast synchronous release and the contribution to AR was later studied in cerebellar and hippocampal synapses (Carter et al., 2002). In both preparations, caffeine-induced Ca^2+^ release from intracellular stores could be efficiently blocked by ryanodine or thapsigargin application. However, neither ryanodine nor thapsigargin had any effect on paired pulse facilitation in cerebellar parallel fibers or in most hippocampal excitatory synapses (Schaffer collaterals, associated commissural input, and mossy fiber input to pyramidal cells). In addition, both drugs failed to block AR evoked by stimulation of parallel fibers. Thus, additional experiments are necessary to determine the impact of Ca^2+^ release from presynaptic intracellular stores on AR generation.

A very interesting hypothesis was proposed by Few et al. (2012). They showed that prolonged or repetitive activation of N- and/or P-types of VGCC triggers sustained Ca^2+^-dependent activation of these channels resulting in long-lasting Ca^2+^ influx. Indeed, this current might be sufficient to trigger vesicle fusion. However, since the peak amplitude of the Ca^2+^-induced current is about 10% of the peak amplitude of the depolarization-induced Ca^2+^ current, the amplitude of asynchronous events should be substantially smaller than that of synchronous fast responses. This is probably the case at synapses formed by cerebellar parallel fibers, but seems to be unlikely in the case of AR from hippocampal CCK-positive basket cells. In the latter connection, the integral of asynchronously released IPSC detected after the bust of Aps is in the same range as the cumulative phasic response evoked during the AP train (Hefft and Jonas, 2005), suggesting similarity in release probability and [Ca^2+^]_i_ during the synchronous and asynchronous phases of release. Even when taking into account the difference in the cooperativity of synchronous and AR (approximately 2–4 fold) and high Ca^2+^ affinity of Syt7, the Ca^2+^-induced tail current through N- and P- type Ca^2+^ channels is unlikely to be sufficient to trigger AR at CCK-positive synapses. In addition to that, activation of CB1 receptors expressed on these terminals leads to suppression of VGCC reducing overall Ca^2+^ entry; this mostly affects synchronous release and has a weaker effect on the asynchronous component (Ali and Todorova, 2010). A similar picture was observed in the zebrafish neuromuscular junction, where blockade of voltage-gated P/Q Ca^2+^ channels during a burst of APs did not prevent either delayed [Ca^2+^]_i_ increase or AR (Wen et al., 2013). Taken together, the findings made by Ali and Todorova (2010) and Wen et al. (2013) suggest that a burst of APs may trigger some additional processes except from Ca^2+^ entry *via* VGCC which may induce [Ca^2+^]_i_ increase and trigger AR.

## Hypothetical Role of Calcium Extrusion in Asynchronous Release

**Figure 3:**
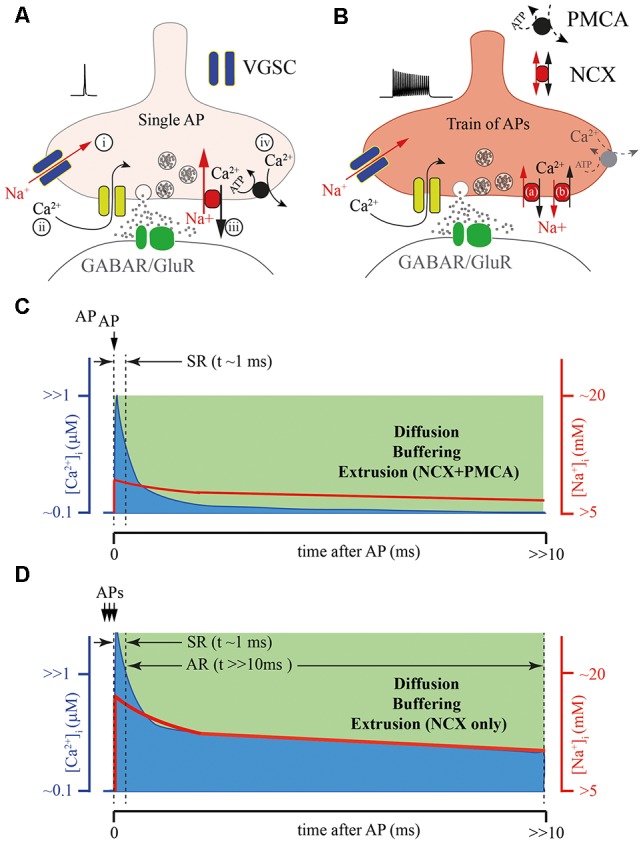
Possible role of the presynaptic calcium extrusion pumps in AR generation.** (A)** Schematic drawing of presynaptic sequence of Na^+^ and Ca^2+^ fluxes triggered by a single AP: (i) Na^+^ entry trough voltage-gated sodium channels (VGSCs); (ii) Ca^2+^ entry trough VGCC; (iii) Fast Ca^2+^ extrusion *via* sodium/calcium exchanger (NCX); and (iv) Final clearance of the presynaptic Ca^2+^ by plasma membrane calcium-ATPase (PMCA). **(B)** Massive elevation of intra-terminal Na^+^ concentration during the high frequency train of APs can strongly reduce the NCX extrusion rate (a), or at extreme elevation of [Na^+^]_i_, reverse the direction of Na^+^ and Ca^2+^ fluxes (b) through NCX prolonging the time course of the presynaptic calcium clearance, especially in terminals with reduced function of PMCA. **(C)** Schematic representation of intraterminal [Ca^2+^]_i_ (blue) and [Na^+^]_i_ (red) time courses after a single AP. **(D)** Schematic representation of the [Ca^2+^]_i_ time course (blue) after burst APs leading to the massive elevation [Na^+^]_i_ (red) when NCX is the only extrusion pump. Note, that direct simultaneous measurements of intraterminal [Na^+^]i and [Ca^2+^]_i_ are not technically possible at the moment. However, modeling studies suggest that in the case of PMCA absence the temporal dynamics of [Ca^2+^]_i_ and [Na^+^]_i_ are be tightly coupled.

Most of the mechanisms of [Ca^2+^]_i_ elevation discussed above, which trigger AR, consider the participation of either ligand-gated or voltage-gated slow Ca^2+^ conductances (Figure 2B). However, taking into account the fact that Syt7-mediated release can be triggered by [Ca^2+^]_i_ in the range of 1 μM, the role of residual Ca^2+^ in AR generation has to be considered. Disruption of Ca^2+^ extrusion from presynaptic terminals might lead to a prolongation of [Ca^2+^]_i_ transients and consequently evoke delayed vesicle fusion. Two major plasma membrane transport proteins are involved in the maintenance of presynaptic Ca^2+^ homeostasis, these are: plasma membrane calcium-ATPase (PMCA) and the sodium/calcium exchanger (NCX; Figures 2A, 3A,C). It was suggested that the major role of PMCA is the maintenance of low cytosolic Ca^2+^, since its affinity to Ca^2+^ is rather high and the rate of extrusion was thought to be slow. In contrast, NCX can rapidly counteract large cytosolic Ca^2+^ elevations especially in excitable cells. However, recently the roles of the two Ca^2+^ extrusion systems have been revised, since it has been shown that some PMCA isoforms may be involved in the regulation of basal Ca^2+^ concentration (in the 100 nM range) and in the Ca^2+^ elevations generated by cell stimulation (in the μM range). For instance, PMCA2, in particular, PMCA2a, exhibits exceptionally rapid activation in response to a rise in [Ca^2+^]_i_ (Caride et al., 2001). PMCA2a is ideally suitable for quick Ca^2+^ handling even during prolonged high-frequency firing. Interestingly, in hippocampal perisomatic inhibitory synapses this isoform is selectively expressed in parvalbumin-containing terminals (Jensen et al., 2007; Burette et al., 2009), while in CCK-terminals characterized by massive AR PMCA2a has not been detected.

NCX is a plasma membrane transport protein that exchanges 3 Na^+^ for 1 Ca^2+^; its functioning is strongly dependent on Na^+^ and Ca^2+^ gradients and plasma membrane potential. Thus, strong Na^+^ accumulation in the cytosol (for example, after a train of APs) substantially slows down NCX-mediated Ca^2+^ extrusion resulting in elevation of residual [Ca^2+^]_i_. Presynaptic Na^+^ dynamics are not well studied, however, several lines of evidence suggest that the decay time constant of Na^+^ extrusion is in the range of hundreds of milliseconds (Regehr, 1997; Fleidervish et al., 2010). Thus, during a high frequency burst of APs Na^+^ concentration can rapidly build up in the terminal and then slowly decay to the basal level; in this period NCX will extrude Ca^2+^ at a substantially slower rate (Figures 3B,D). Elevation of residual [Ca^2+^]_i_ due to Na^+^-dependent decelerating of NCX-mediated Ca^2+^ extrusion is even more pronounced at synapses with reduced PMCA function (Roome et al., 2013a). Moreover, extreme elevation of [Na^+^]_i_ may reverse NCX and result in Ca^2+^ influx into the cell *via* this exchanger (Roome et al., 2013b; Khananshvili, 2014). The latter suggests that NCX may act either as a Ca^2+^-clearing protein or Ca^2+^ source, depending on the intensity of presynaptic activity (Figure 3A). In the case of CCK-positive hippocampal basket cells, which do not express the fast isoform of PMCA, slowing of the rate of NCX-mediated extrusion or switching to NCX reverse mode might provide a level of [Ca^2+^]_i_ sufficient for AR generation. Selective suppression of AR at these GABAergic synapses and at some excitatory terminals by moderate concentration of EGTA strongly suggests that residual Ca^2+^ and Ca^2+^ extrusion machinery, determining the kinetics of [Ca^2+^]_i_ are involved in delayed release generation (Hefft and Jonas, 2005; Iremonger and Bains, 2007).

## Asynchronous Release Triggered by Strontium or Lanthanides

Substitution of extracellular Ca^2+^ with Sr^2+^ or application of lanthanides reduces phasic release and greatly promotes AR (Dodge et al., 1969; Heuser and Miledi, 1971; Goda and Stevens, 1994; Xu-Friedman and Regehr, 2000; Shin et al., 2003). Although, the underlying mechanisms are certainly different from AR evoked at physiological conditions by high frequency stimulation, some of the effects of Sr^2+^ and La^3+^ on the timing of synaptic release can be explained by a reduction in the functioning of extrusion pumps.

Strontium can enter the terminals and trigger synaptic vesicle fusion *via* interaction with Syt1, although in a way that does not involve activation of SNARE (Shin et al., 2003; Li et al., 2017). However, in contrast to Ca^2+^, clearance of “residual Sr^2+^” from presynaptic terminals is significantly slower (Xu-Friedman and Regehr, 2000), which can explain the extended time course of Sr^2+^-driven release. The rapid effect of La^3+^ does not require La^3+^ entry into the terminal, or binding to Syt1 and is independent of extracellular Ca^2+^ concentration. Nevertheless, the delayed component of an La^3+^-evoked increase of spontaneous release frequency can be blocked by intracellular loading of Ca^2+^ buffers (Chung et al., 2008). The latter, probably, can be attributed to the known ability of La^3+^ to block PMCA (Shimizu et al., 1997) which results in elevation of [Ca^2+^]_i_ and promotes vesicle fusion. Thus, one can assume that the lack or reduced function of one of the extrusion proteins may result in slowed presynaptic [Ca^2+^]_i_ dynamics leading to prolongation of vesicle release.

## Concluding Remarks

Currently, there is an agreement that the Ca^2+^ sensors involved in fast and AR are different and that they have different Ca^2+^-binding kinetics. Syt7 has been proposed to perform the function of high affinity Ca^2+^ sensors for AR generation that is spatially and temporally located outside Ca^2+^ domains. However, Syt7 was found on the presynaptic plasma membrane and other internal membranes, but not on synaptic vesicles suggesting a non-canonical mechanism of Syt7-mediated of vesicle exocytosis. Thus, recruitment of Syt7 into evoked delayed release needs to be more thoroughly studied. In addition to that, involvement of other synaptotagmins to AR generation, which have high Ca^2+^ affinity and neuronal expression, has to be investigated. Importantly, experiments with EGTA loading clearly show that AR requires the presence of long-lasting elevation of free intraterminal Ca^2+^. In this respect, it might be promising to study the possible role of modulation of Ca^2+^ extrusion proteins. The role of NCX should be investigated, since the NCX-mediated extrusion rate depends on [Na^2+^]i, which is determined by the rate of presynaptic AP activity. Thus, Na^+^ dependent modulation of NCX functioning might provide an alternative mechanism not only for AR generation but also for short-term plasticity.

## Author Contributions

All authors listed have made substantial, direct, and intellectual contribution to the work and approved it for publication.

## Conflict of Interest Statement

The authors declare that the research was conducted in the absence of any commercial or financial relationships that could be construed as a potential conflict of interest.

## References

[B1] Ali A. B. Todorova M. (2010). Asynchronous release of GABA via tonic cannabinoid receptor activation at identified interneuron synapses in rat CA1. Eur. J. Neurosci. 31, 1196–1207. 10.1111/j.1460-9568.2010.07165.x 20345910

[B2] Arai I. Jonas P. (2014). Nanodomain coupling explains Ca^2+^ independence of transmitter release time course at a fast central synapse. Elife 3:e04057. 10.7554/elife.04057 25487988 PMC4270082

[B3] Bacaj T. Wu D. Yang X. Morishita W. Zhou P. Xu W.. (2013). Synaptotagmin-1 and synaptotagmin-7 trigger synchronous and asynchronous phases of neurotransmitter release. Neuron 80, 947–959. 10.1016/j.neuron.2013.10.026 24267651 PMC3888870

[B4] Bhalla A. Chicka M. C. Chapman E. R. (2008). Analysis of the synaptotagmin family during reconstituted membrane fusion. J. Biol. Chem. 283, 21799–21807. 10.1074/jbc.M709628200 18508778 PMC2490792

[B5] Burette A. C. Strehler E. E. Weinberg R. J. (2009). “Fast” plasma membrane calcium pump PMCA2a concentrates in GABAergic terminals in the adult rat brain. J. Comp. Neurol. 512, 500–513. 10.1002/cne.21909 19025983 PMC2635118

[B6] Burkitt A. N. Clark G. M. (1999). Analysis of integrate-and-fire neurons: synchronization of synaptic input and spike output. Neural Comput. 11, 871–901. 10.1162/089976699300016485 10226187

[B7] Caride A. J. Penheiter A. R. Filoteo A. G. Bajzer Z. Enyedi A. Penniston J. T. (2001). The plasma membrane calcium pump displays memory of past calcium spikes. Differences between isoforms 2b and 4b. J. Biol. Chem. 276, 39797–39804. 10.1074/jbc.M104380200 11514555

[B8] Carter A. G. Vogt K. E. Foster K. A. Regehr W. G. (2002). Assessing the role of calcium-induced calcium release in short-term presynaptic plasticity at excitatory central synapses. J. Neurosci. 22, 21–28. 10.1523/JNEUROSCI.22-01-00021.2002 11756484 PMC6757598

[B9] Cembrowski M. S. Bachman J. L. Wang L. Sugino K. Shields B. C. Spruston N. (2016). Spatial gene-expression gradients underlie prominent heterogeneity of CA1 pyramidal neurons. Neuron 89, 351–368. 10.1016/j.neuron.2015.12.013 26777276

[B10] Chad J. E. Eckert R. (1984). Calcium domains associated with individual channels can account for anomalous voltage relations of CA-dependent responses. Biophys. J. 45, 993–999. 10.1016/s0006-3495(84)84244-7 6329349 PMC1434976

[B11] Chen C. Satterfield R. Young S. M. Jr. Jonas P. (2017). Triple function of synaptotagmin 7 ensures efficiency of high-frequency transmission at central GABAergic synapses. Cell Rep. 21, 2082–2089. 10.1016/j.celrep.2017.10.122 29166601 PMC5863544

[B12] Chung C. Deak F. Kavalali E. T. (2008). Molecular substrates mediating lanthanide-evoked neurotransmitter release in central synapses. J. Neurophysiol. 100, 2089–2100. 10.1152/jn.90404.2008 18715899 PMC2576212

[B13] Courtney N. A. Briguglio J. S. Bradberry M. M. Greer C. Chapman E. R. (2018). Excitatory and inhibitory neurons utilize different Ca^2+^ sensors and sources to regulate spontaneous release. Neuron 98, 977–991. 10.1016/j.neuron.2018.04.022 29754754 PMC6090561

[B14] Craxton M. (2010). A manual collection of Syt, Esyt, Rph3a, Rph3al, Doc2, and Dblc2 genes from 46 metazoan genomes—an open access resource for neuroscience and evolutionary biology. BMC Genomics 11:37. 10.1186/1471-2164-11-37 20078875 PMC2823689

[B15] Daw M. I. Tricoire L. Erdelyi F. Szabo G. McBain C. J. (2009). Asynchronous transmitter release from cholecystokinin-containing inhibitory interneurons is widespread and target-cell independent. J. Neurosci. 29, 11112–11122. 10.1523/JNEUROSCI.5760-08.2009 19741117 PMC2762613

[B16] Dodge F. A. Jr. Miledi R. Rahamimoff R. (1969). Strontium and quantal release of transmitter at the neuromuscular junction. J. Physiol. 200, 267–283. 10.1113/jphysiol.1969.sp008692 4387376 PMC1350428

[B17] Eggermann E. Bucurenciu I. Goswami S. P. Jonas P. (2011). Nanodomain coupling between Ca^2+^ channels and sensors of exocytosis at fast mammalian synapses. Nat. Rev. Neurosci. 13, 7–21. 10.1038/nrn3125 22183436 PMC3617475

[B18] Few A. P. Nanou E. Watari H. Sullivan J. M. Scheuer T. Catterall W. A. (2012). Asynchronous Ca^2+^ current conducted by voltage-gated Ca^2+^ (Ca_V_)-2.1 and Ca_V_2.2 channels and its implications for asynchronous neurotransmitter release. Proc. Natl. Acad. Sci. U S A 109, E452–E460. 10.1073/pnas.1121103109 22308469 PMC3289374

[B19] Fleidervish I. A. Lasser-Ross N. Gutnick M. J. Ross W. N. (2010). Na^+^ imaging reveals little difference in action potential-evoked Na^+^ influx between axon and soma. Nat. Neurosci. 13, 852–860. 10.1038/nn.2574 20543843 PMC3102307

[B20] Fukuda M. Kanno E. Satoh M. Saegusa C. Yamamoto A. (2004). Synaptotagmin VII is targeted to dense-core vesicles and regulates their Ca^2+^ -dependent exocytosis in PC12 cells. J. Biol. Chem. 279, 52677–52684. 10.1074/jbc.m409241200 15456748

[B21] Geppert M. Goda Y. Hammer R. E. Li C. Rosahl T. W. Stevens C. F.. (1994). Synaptotagmin I: a major Ca^2+^ sensor for transmitter release at a central synapse. Cell 79, 717–727. 10.1016/0092-8674(94)90556-8 7954835

[B22] Goda Y. Stevens C. F. (1994). Two components of transmitter release at a central synapse. Proc. Natl. Acad. Sci. U S A 91, 12942–12946. 10.1073/pnas.91.26.12942 7809151 PMC45556

[B23] Groffen A. J. Martens S. Díez A. R. Cornelisse L. N. Lozovaya N. de Jong A. P.. (2010). Doc2b is a high-affinity Ca^2+^ sensor for spontaneous neurotransmitter release. Science 327, 1614–1618. 10.1126/science.1183765 20150444 PMC2846320

[B24] Gustavsson N. Han W. (2009). Calcium-sensing beyond neurotransmitters: functions of synaptotagmins in neuroendocrine and endocrine secretion. Biosci. Rep. 29, 245–259. 10.1042/bsr20090031 19500075

[B25] Gustavsson N. Lao Y. Maximov A. Chuang J. C. Kostromina E. Repa J. J.. (2008). Impaired insulin secretion and glucose intolerance in synaptotagmin-7 null mutant mice. Proc. Natl. Acad. Sci. U S A 105, 3992–3997. 10.1073/pnas.0711700105 18308938 PMC2268794

[B26] Hefft S. Jonas P. (2005). Asynchronous GABA release generates long-lasting inhibition at a hippocampal interneuron-principal neuron synapse. Nat. Neurosci. 8, 1319–1328. 10.1038/nn1542 16158066

[B27] Heuser J. Miledi R. (1971). Effects of lanthanum ions on function and structure of frog neuromuscular junctions. Proc. R. Soc. Lond. B Biol. Sci. 179, 247–260. 10.1098/rspb.1971.0096 4400214

[B28] Iremonger K. J. Bains J. S. (2007). Integration of asynchronously released quanta prolongs the postsynaptic spike window. J. Neurosci. 27, 6684–6691. 10.1523/JNEUROSCI.0934-07.2007 17581955 PMC6672686

[B29] Jackman S. L. Turecek J. Belinsky J. E. Regehr W. G. (2016). The calcium sensor synaptotagmin 7 is required for synaptic facilitation. Nature 529, 88–91. 10.1038/nature16507 26738595 PMC4729191

[B30] Jappy D. Valiullina F. Draguhn A. Rozov A. (2016). GABA_B_R-dependent long-term depression at hippocampal synapses between CB1-positive interneurons and CA1 pyramidal cells. Front. Cell. Neurosci. 10:4. 10.3389/fncel.2016.00004 26858602 PMC4729905

[B31] Jensen T. P. Filoteo A. G. Knopfel T. Empson R. M. (2007). Presynaptic plasma membrane Ca^2+^ ATPase isoform 2a regulates excitatory synaptic transmission in rat hippocampal CA3. J. Physiol. 579, 85–99. 10.1113/jphysiol.2006.123901 17170045 PMC2075377

[B32] Kaeser P. S. Regehr W. G. (2014). Molecular mechanisms for synchronous, asynchronous, and spontaneous neurotransmitter release. Annu. Rev. Physiol. 76, 333–363. 10.1146/annurev-physiol-021113-170338 24274737 PMC4503208

[B33] Khakh B. S. (2009). ATP-gated P2X receptors on excitatory nerve terminals onto interneurons initiate a form of asynchronous glutamate release. Neuropharmacology 56, 216–222. 10.1016/j.neuropharm.2008.06.011 18601937

[B34] Khananshvili D. (2014). Sodium-calcium exchangers (NCX): molecular hallmarks underlying the tissue-specific and systemic functions. Pflugers Arch. 466, 43–60. 10.1007/s00424-013-1405-y 24281864

[B35] Klausberger T. Somogyi P. (2008). Neuronal diversity and temporal dynamics: the unity of hippocampal circuit operations. Science 321, 53–57. 10.1126/science.1149381 18599766 PMC4487503

[B37] Li Y. C. Chanaday N. L. Xu W. Kavalali E. T. (2017). Synaptotagmin-1- and synaptotagmin-7-dependent fusion mechanisms target synaptic vesicles to kinetically distinct endocytic pathways. Neuron 93, 616.e3–631.e3. 10.1016/j.neuron.2016.12.010 28111077 PMC5300960

[B36] Li J. Xiao Y. Zhou W. Wu Z. Zhang R. Xu T. (2009). Silence of Synaptotagmin VII inhibits release of dense core vesicles in PC12 cells. Sci. China C Life Sci. 52, 1156–1163. 10.1007/s11427-009-0160-y 20016973

[B38] Liu H. Bai H. Hui E. Yang L. Evans C. S. Wang Z.. (2014). Synaptotagmin 7 functions as a Ca^2+^-sensor for synaptic vesicle replenishment. Elife 3:e01524. 10.7554/elife.01524 24569478 PMC3930910

[B39] Luo F. Sudhof T. C. (2017). Synaptotagmin-7-mediated asynchronous release boosts high-fidelity synchronous transmission at a central synapse. Neuron 94, 826–839. 10.1016/j.neuron.2017.04.020 28521135

[B40] Maximov A. Lao Y. Li H. Chen X. Rizo J. Sørensen J. B.. (2008). Genetic analysis of synaptotagmin-7 function in synaptic vesicle exocytosis. Proc. Natl. Acad. Sci. U S A 105, 3986–3991. 10.1073/pnas.0712372105 18308933 PMC2268828

[B41] Moghadam P. K. Jackson M. B. (2013). The functional significance of synaptotagmin diversity in neuroendocrine secretion. Front. Endocrinol. 4:124. 10.3389/fendo.2013.00124 24065953 PMC3776153

[B42] Narita K. Akita T. Hachisuka J. Huang S. Ochi K. Kuba K. (2000). Functional coupling of Ca^2+^ channels to ryanodine receptors at presynaptic terminals. J. Gen. Physiol. 115, 519–532. 10.1085/jgp.115.4.519 10736317 PMC2233761

[B43] Narita K. Akita T. Osanai M. Shirasaki T. Kijima H. Kuba K. (1998). A Ca^2+^-induced Ca^2+^ release mechanism involved in asynchronous exocytosis at frog motor nerve terminals. J. Gen. Physiol. 112, 593–609. 10.1085/jgp.112.5.593 9806968 PMC2229444

[B44] Neher E. (1998). Vesicle pools and Ca^2+^ microdomains: new tools for understanding their roles in neurotransmitter release. Neuron 20, 389–399. 10.1016/s0896-6273(00)80983-6 9539117

[B45] Pang Z. P. Bacaj T. Yang X. Zhou P. Xu W. Südhof T. C. (2011). Doc2 supports spontaneous synaptic transmission by a Ca^2+^-independent mechanism. Neuron 70, 244–251. 10.1016/j.neuron.2011.03.011 21521611 PMC3102832

[B46] Pang Z. P. Shin O. H. Meyer A. C. Rosenmund C. Südhof T. C. (2006). A gain-of-function mutation in synaptotagmin-1 reveals a critical role of Ca^2+^-dependent soluble N-ethylmaleimide-sensitive factor attachment protein receptor complex binding in synaptic exocytosis. J. Neurosci. 26, 12556–12565. 10.1523/JNEUROSCI.3804-06.2006 17135417 PMC6674888

[B47] Ramirez D. M. O. Crawford D. C. Chanaday N. L. Trauterman B. Monteggia L. M. Kavalali E. T. (2017). Loss of Doc2-dependent spontaneous neurotransmission augments glutamatergic synaptic strength. J. Neurosci. 37, 6224–6230. 10.1523/JNEUROSCI.0418-17.2017 28539418 PMC5490061

[B48] Regehr W. G. (1997). Interplay between sodium and calcium dynamics in granule cell presynaptic terminals. Biophys. J. 73, 2476–2488. 10.1016/s0006-3495(97)78276-6 9370441 PMC1181149

[B49] Roome C. J. Knöpfel T. Empson R. M. (2013a). Functional contributions of the plasma membrane calcium ATPase and the sodium-calcium exchanger at mouse parallel fibre to Purkinje neuron synapses. Pflugers Arch. 465, 319–331. 10.1007/s00424-012-1172-1 23138229

[B50] Roome C. J. Power E. M. Empson R. M. (2013b). Transient reversal of the sodium/calcium exchanger boosts presynaptic calcium and synaptic transmission at a cerebellar synapse. J. Neurophysiol. 109, 1669–1680. 10.1152/jn.00854.2012 23255722

[B51] Schonn J. S. Maximov A. Lao Y. Südhof T. C. Sørensen J. B. (2008). Synaptotagmin-1 and -7 are functionally overlapping Ca^2+^ sensors for exocytosis in adrenal chromaffin cells. Proc. Natl. Acad. Sci. U S A 105, 3998–4003. 10.1073/pnas.0712373105 18308932 PMC2268838

[B52] Segovia M. Ales E. Montes M. A. Bonifas I. Jemal I. Lindau M.. (2010). Push-and-pull regulation of the fusion pore by synaptotagmin-7. Proc. Natl. Acad. Sci. U S A 107, 19032–19037. 10.1073/pnas.1014070107 20956309 PMC2973888

[B53] Shimizu H. Borin M. L. Blaustein M. P. (1997). Use of La^3+^ to distinguish activity of the plasmalemmal Ca^2+^ pump from Na^+^/Ca^2+^ exchange in arterial myocytes. Cell Calcium 21, 31–41. 10.1016/s0143-4160(97)90094-4 9056075

[B54] Shin O. H. Rhee J. S. Tang J. Sugita S. Rosenmund C. Südhof T. C. (2003). Sr^2+^ binding to the Ca^2+^ binding site of the synaptotagmin 1 C2B domain triggers fast exocytosis without stimulating SNARE interactions. Neuron 37, 99–108. 10.1016/s0896-6273(02)01145-5 12526776

[B55] Shin O. H. Rizo J. Südhof T. C. (2002). Synaptotagmin function in dense core vesicle exocytosis studied in cracked PC12 cells. Nat. Neurosci. 5, 649–656. 10.1038/nn869 12055633

[B56] Simon S. M. Llinás R. R. (1985). Compartmentalization of the submembrane calcium activity during calcium influx and its significance in transmitter release. Biophys. J. 48, 485–498. 10.1016/s0006-3495(85)83804-2 2412607 PMC1329362

[B57] Südhof T. C. (2013). Neurotransmitter release: the last millisecond in the life of a synaptic vesicle. Neuron 80, 675–690. 10.1016/j.neuron.2013.10.022 24183019 PMC3866025

[B58] Sugita S. Han W. Butz S. Liu X. Fernández-Chacón R. Lao Y.. (2001). Synaptotagmin VII as a plasma membrane Ca^2+^ sensor in exocytosis. Neuron 30, 459–473. 10.1016/s0896-6273(01)00290-2 11395007

[B59] Sugita S. Shin O. H. Han W. Lao Y. Südhof T. C. (2002). Synaptotagmins form a hierarchy of exocytotic Ca^2+^ sensors with distinct Ca^2+^ affinities. EMBO J. 21, 270–280. 10.1093/emboj/21.3.270 11823420 PMC125835

[B60] Sun J. Pang Z. P. Qin D. Fahim A. T. Adachi R. Südhof T. C. (2007). A dual-Ca^2+^-sensor model for neurotransmitter release in a central synapse. Nature 450, 676–682. 10.1038/nature06308 18046404 PMC3536472

[B61] Takamori S. Holt M. Stenius K. Lemke E. A. Grønborg M. Riedel D.. (2006). Molecular anatomy of a trafficking organelle. Cell 127, 831–846. 10.1016/j.cell.2006.10.030 17110340

[B62] Tsuboi T. Fukuda M. (2007). Synaptotagmin VII modulates the kinetics of dense-core vesicle exocytosis in PC12 cells. Genes Cells 12, 511–519. 10.1111/j.1365-2443.2007.01070.x 17397398

[B63] Turecek J. Regehr W. G. (2018). Synaptotagmin 7 mediates both facilitation and asynchronous release at granule cell synapses. J. Neurosci. 38, 3240–3251. 10.1523/jneurosci.3207-17.2018 29593071 PMC5884459

[B64] Virmani T. Han W. Liu X. Sudhof T. C. Kavalali E. T. (2003). Synaptotagmin 7 splice variants differentially regulate synaptic vesicle recycling. EMBO J. 22, 5347–5357. 10.1093/emboj/cdg514 14532108 PMC213769

[B65] Watanabe J. Rozov A. Wollmuth L. P. (2005). Target-specific regulation of synaptic amplitudes in the neocortex. J. Neurosci. 25, 1024–1033. 10.1523/jneurosci.3951-04.2005 15673684 PMC6725631

[B66] Weber J. P. Toft-Bertelsen T. L. Mohrmann R. Delgado-Martinez I. Sørensen J. B. (2014). Synaptotagmin-7 is an asynchronous calcium sensor for synaptic transmission in neurons expressing SNAP-23. PLoS One 9:e114033. 10.1371/journal.pone.0114033 25422940 PMC4244210

[B67] Wen H. Hubbard J. M. Rakela B. Linhoff M. W. Mandel G. Brehm P. (2013). Synchronous and asynchronous modes of synaptic transmission utilize different calcium sources. Elife 2:e01206. 10.7554/elife.01206 24368731 PMC3869123

[B68] Wen H. Linhoff M. W. McGinley M. J. Li G. L. Corson G. M. Mandel G.. (2010). Distinct roles for two synaptotagmin isoforms in synchronous and asynchronous transmitter release at zebrafish neuromuscular junction. Proc. Natl. Acad. Sci. U S A 107, 13906–13911. 10.1073/pnas.1008598107 20643933 PMC2922265

[B69] Xu J. Mashimo T. Südhof T. C. (2007). Synaptotagmin-1, -2 and -9: Ca^2+^ sensors for fast release that specify distinct presynaptic properties in subsets of neurons. Neuron 54, 567–581. 10.1016/j.neuron.2007.05.004 17521570

[B70] Xue R. Gaffaney J. D. Chapman E. R. (2015). Structural elements that underlie Doc2β function during asynchronous synaptic transmission. Proc. Natl. Acad. Sci. U S A 112, E4316–E4325. 10.1073/pnas.1502288112 26195798 PMC4534243

[B71] Xu-Friedman M. A. Regehr W. G. (2000). Probing fundamental aspects of synaptic transmission with strontium. J. Neurosci. 20, 4414–4422. 10.1523/jneurosci.20-12-04414.2000 10844010 PMC6772435

[B72] Yao J. Gaffaney J. D. Kwon S. E. Chapman E. R. (2011). Doc2 is a Ca^2+^ sensor required for asynchronous neurotransmitter release. Cell 147, 666–677. 10.1016/j.cell.2011.09.046 22036572 PMC3220409

[B73] Zeisel A. Hochgerner H. Lonnerberg P. Johnsson A. Memic F. van der Zwan J.. (2018). Molecular architecture of the mouse nervous system. Cell 174, 999.e22–1014.e22. 10.1016/j.cell.2018.06.021 30096314 PMC6086934

[B74] Zeisel A. Muñoz-Manchado A. B. Codeluppi S. Lönnerberg P. La M. G. Juréus A.. (2015). Brain structure. Cell types in the mouse cortex and hippocampus revealed by single-cell RNA-seq. Science 347, 1138–1142. 10.1126/science.aaa1934 25700174

